# GCA: Better ✓check for third cranial nerve involvement!

**DOI:** 10.1007/s00330-021-07739-7

**Published:** 2021-03-13

**Authors:** Daniel Schwarz

**Affiliations:** Department of Neuroradiology, Heidelberg University Hospital, INF 400, 69120 Heidelberg, Germany

## Abstract

*• The intriguing “Check Mark Sign” suggests 3rd cranial nerve involvement in GCA.*

Giant cell arteritis (GCA) is the most frequent systemic vasculitis of medium-sized to larger blood vessels, typically affecting individuals over the age of 50 years.

While biopsy still represents the gold standard in diagnosing GCA, high-resolution magnetic resonance imaging (HR MRI) has increasingly shown potential for non-invasive diagnosis of this condition with numerous studies reporting high sensitivity and specificity [1, 2]. This has recently prompted the European League Against Rheumatism to rank HR MRI as a first-line imaging tool with the main advantages being high standardization of data acquisition, reduced probability to miss positive findings in case of skip lesions, and possible detection of intracranial manifestations [3].

Co-involvement of the visual and oculomotor systems may be seen in up to 20% of cases in GCA potentially leading to a number of severe complications such as anterior ischemic optic neuropathy [4]. While HR MRI has already proven valuable in the workup of visual symptoms in GCA, little is known about the usefulness of HR imaging in the setting of oculomotor symptoms like diplopia—which may be due to the fact that direct visualization of cranial nerves has always been highly challenging beyond their cisternal segments.

The current study by Mournet et al [5] is the first to shed light on this delicate issue: In their paper, the authors investigate the presence of intracranial nerve abnormalities in a retrospective cohort of GCA patients with ocular motor involvement using HR MRI. While the overall number of patients assessed is relatively low (14/64 with diplopia, 8/14 with 3^rd^ cranial nerve (CN) impairment), they show an impressive sensitivity in 7/8 patients with positive 3^rd^ CN involvement on post contrast black-blood and high-resolution T2-weighted imaging—the only exception being one patient suffering from transient diplopia. Intriguingly, this 3^rd^ CN involvement is reported to give rise to a specific imaging sign, which the authors refer to as “Check Mark Sign”: A characteristic ✓-shaped enhancement or T2-weighted hyperintensity within the orbital apex that is due to the typical branching of the 3^rd^ CN within the superior orbital fissure. This imaging finding is nicely presented by the authors using instructive examples and a high interrater agreement is achieved.

However, in patients with either 4^th^ or 6^th^ CN impairment, the authors do not observe any nerve involvement on imaging. On the one hand, this is good news because these patients (1/14 with 4^th^ CN and 5/14 with 6^th^ CN impairment) serve as a convincing internal control group underlining a high degree of specificity with regard to the findings affecting the 3^rd^ cranial nerve. On the other hand, it shows that either (a) technical limitations still preclude successful detection of nerve abnormalities or (b) an alternative pathophysiological process is involved that may not be readily depicted.

In a nutshell, the current work by Mournet et al constitutes a valuable contribution to the imaging diagnostics in GCA further extending the radiological prospect to direct cranial nerve involvement. This becomes feasible by high-resolution imaging, especially by the smart use of black-blood imaging. Their findings give rise to a compelling, almost “Aunt Minnie”-type of imaging sign which may also be of interest in other types of orbital pathologies in the future.

## Declarations

### Guarantor

The scientific guarantor of this publication is Daniel Schwarz.

### Statistics and biometry

No complex statistical methods were necessary for this paper.

### Methodology

• Editorial comment
